# Uniplanar Nystagmus Associated with Perceptual and Cognitive Visual Dysfunction due to Presumed Focal Ischemic Occipital Cortical Atrophy: A Missed Diagnosis and New Observation

**DOI:** 10.1155/2012/159746

**Published:** 2012-09-27

**Authors:** Swetha Sara Philip, Gordon N. Dutton, Liam Dorris

**Affiliations:** ^1^Department of Ophthalmology, Christian Medical College and Hospital, Vellore, Tamil Nadu, Vellore 632001, India; ^2^Royal Hospital for Sick Children, Yorkhill, Glasgow G3 8SJ, UK

## Abstract

Uniplanar nystagmus has been described in relation to pathology of the brain stem, retina, optic nerve, sensory visual deprivation, periventricular leucomalacia, and drug toxicity. This paper describes a case of uniplanar nystagmus associated with features of higher visual dysfunction and a presumed focal insult to the occipital lobes following an episode of neonatal apnea.

## 1. Introduction

Uniplanar nystagmus (the pattern of which is in the same plane for each position of gaze) in children occurs in relation to idiopathic brain stem dysfunction [1], retinal disorders of both rods and cones, optic nerve hypoplasia, foveal hypoplasia (which may manifest in albinism), early onset sensory visual deprivation [2], periventricular leucomalacia [3] and drug (methadone) toxicity [4]. We report a case of uniplanar nystagmus associated with evidence of perceptual and cognitive visual dysfunction in a patient with presumed focal insult to the occipital lobes following one episode of neonatal apnea.

## 2. Case Report

An 18-year-old girl with mild learning disability and poor vision was referred to the vision clinic in a tertiary care hospital. She had been born with no complications at 40-weeks gestation by spontaneous vaginal delivery. The pregnancy was uneventful. At two weeks of age, she had an apneic attack at home, possibly due to choking, and was resuscitated by the ambulance service after 10 minutes of cyanosis. Subsequently, she developed “congenital” nystagmus and maintained good health. She wore glasses for astigmatism. She was studying in mainstream school with the use of magnifiers, as she was having difficulty reading. At secondary school, she was given additional educational support on account of her visual difficulties. Her difficulties not only related to vision but also to her interpretation of the visual environment and this caused embarrassment and lack of confidence.

On examination, her visual acuities were right eye 20/80 and left eye 20/60, and 20/40 binocularly for single letters (Snellen's test type). For crowded letters, her binocular visual acuity was 20/80 and N18 for reading material. Extraocular movements were full. There was uniplanar (horizontal) manifest nystagmus with a diminished amplitude (or null point) on slight left gaze. Examination of the eyes was normal. Visual field examination proved difficult unless the patient was prompted, because peripheral visual inattention was evident. No colour vision deficit was identified. 

### 2.1. Structured History Taking [5]

Her mother described that, as a child, the patient bumped into obstacles and was not able to judge when the pavement ended and the road began. She was unable to identify distant targets. She did not like it when the furniture in the house was moved, as she then bumped into it. She had problems finding objects on a patterned background, that is, she could rarely find her clothes on a patterned bedspread. She hated shopping as she could not cope with the crowds. She explained that it was difficult to recognize faces, especially when concentrating on other things. She found it very difficult to identify the family car from among other parked cars.  Table 1(a) summarizes the features elicited. 

### 2.2. Investigations

Binocular VEP to reversing black and white checkerboards (120′ element size) showed delayed P100 recordings (150 ms; upper limit of normal 110 ms). Monocular pattern-onset VEPs showed delayed C1 at 160 ms and 164 ms for the right and left eyes, respectively (upper limit of normal 115 ms). The hemispheric VEP distribution was in keeping with normal chiasmal crossing. Binocular ERGs were normal for photopic and scotopic conditions. These findings render albinism or retinal disorders very unlikely as causes of her nystagmus. 

**Figure 1:**
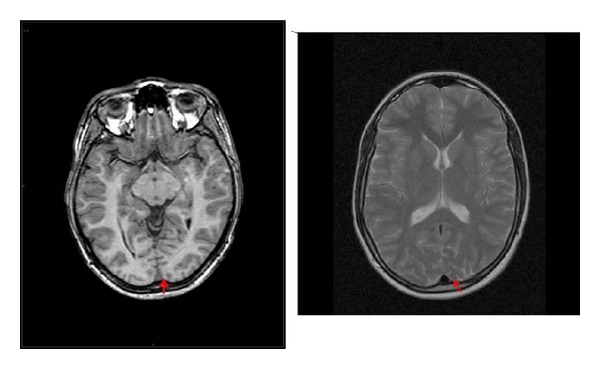
MRI scan of the brain and orbit. The ventricles appear normal. The white matter thickness around the cerebral hemispheres is normal but the grey matter (as shown by red arrows) around the occipital poles is diminished in thickness and poorly defined. The brain folding and myelination are normal.

MRI of the brain showed major structures to be present and intact (Figure 1). The optic nerves, chiasm, and optic tracts showed no abnormality. The corpus callosum and posterior pituitary gland were normal. There was no significant ventricular dilation or asymmetry in the posterior horns of the lateral ventricles and no gross asymmetry of the cerebral hemispheres. The white matter thickness around this region was normal but the grey matter thickness in both occipital poles was diminished and poorly defined, consistent with a cortical atrophy. Brain folding and myelination, apart from the occipital poles, were normal.

**Neuropsychological Testing**

The patient was assessed using the Wechsler Adult Intelligence Scale (WAIS-III UK edition) and had an overall IQ within the higher end of the “mild learning disability” range (Verbal IQ = 65, Performance IQ = 70); the Visual Object and Space Perception Test (VOSP) revealed particular problems in tests of space perception (Table 1(b)). These deficits accorded with her self-report of reading and writing difficulties, a significant visuoperceptual disorder, and difficulty in independently navigating novel environments. The developmental impact of her cognitive difficulties was also felt to be a major factor in the aetiology of a number of psychological disorders including clinically significant anxiety disorder.

## 3. Discussion

Higher visual processing takes place in areas adjacent to the occipital cortex: the ventral stream connects the occipital cortex to the temporal lobes and provides conscious visual percepts for “offline” perception and control of action; the dorsal stream connects the occipital lobes to the posterior parietal lobes and provides vision for action for “online” control of action [6, 7]. Damage to the ventral stream and the dorsal stream leads to disorders of visual perception and guidance of movement, that may only come to light when a child reaches school age [8].

This case exhibits features that we believe to be hitherto unreported. The perceptual visual difficulties are typical of cerebral visual impairment (CVI) affecting visual acuities and functions served by the dorsal and ventral streams [7]. CVI is a silent cause for visual impairment of increasing incidence in children in the developed and developing world due to improvement in neonatal and pediatric care [9]. Perinatal hypoxic-ischaemic brain injury is said to be the most common cause [10] in both preterm and term infants. The sequelae of the hypoxic-ischaemic episode depend on the gestational age of the child [11]. It is estimated that about 60% of children with hypoxic-ischemic brain injury have CVI [12]. The pathogenesis has yet to be understood fully but relates to damage to the parasaggital watershed zones affecting the periventricular white matter in premature infants and infarction of the striate cortex in full-term babies [13].

Refractive error, poor accommodation [14], strabismus, nystagmus, optic atrophy [9], and lower visual field loss are seen in children with CVI, along with simultanagnosia and optic ataxia [15]. Children with CVI due to periventricular white matter injury present with nystagmus that has different waveforms and latencies [3]. The exact cause of nystagmus in CVI is still uncertain. It has been postulated that either there could be a disruption of visual processing from the occipital cortex to the parietal and frontal cortices or dorsal stream pathway dysfunction may be the cause. This pathway is affected in CVI and is associated with impaired visual perception [3].

In a healthy neonate, severe asphyxia of less than 10 minutes may not cause any permanent damage to the brain but can present with cranial nerve dysfunction or athetoid cerebral palsy and show up on MRI with tissue loss and limited signal changes [16]. Focal occipital cortical atrophy due to presumed hypoxic damage in infancy has previously been described [16] but not the clinical features. Uniplanar early onset nystagmus is thought not to be a feature of cortical pathology. Not surprisingly we could find no publications concerning this putative lack of association. However, we have identified a similar reported case with such cortical atrophy in the absence of spontaneous nystagmus [17].

The perceptual visual difficulties in this young lady went undiagnosed for the first 18 years of her life and it was not until detailed structured history taking revealed perceptual visual dysfunction that imaging was carried out, the diagnosis made and targeted appropriate intervention instigated [5]. Such pathology, with the typical clinical features of perceptual and cognitive visual dysfunction, has not to our knowledge hitherto been described. The association between focal occipital cortical damage and the nystagmus could either be indirect (with the potential for additional focal pathology causing nystagmus) or direct. Thus notwithstanding whether the nystagmus is directly related to the cortical pathology or is an epiphenomenon, this case highlights the importance of taking a good history for cognitive and perceptual visual impairment as recommended by a number of authors [18–20] for early diagnosis and implementation of appropriate care.

## Figures and Tables

**Table 1a:** (a) Perceptual visual difficulties described by the patient

Features	Problems
Features of dorsal stream dysfunction	

Impaired ability to handle complex visual scenes	Drop in visual acuity for crowded letters
	Tendency to get lost in crowded location
	Difficulty to see things on patterned background
	Cannot cope with supermarkets
	Cannot see things pointed out in the distance
	Need to view television from a distance of 30 cms

Impaired visually guided movement	Negotiating steps and kerbs
	Cannot cross roads
	Bumping into objects
	Hates going out at night
	Intensely dislike furniture being moved because she then bumps into it

Impaired attention	Cannot recognize faces when concentrating on other things

Features of ventral stream dysfunction	

Impaired recognition	Cannot differentiate between different makes of cars
	Has difficulty recognizing faces

**Table 1b:** (b) Visual object and space perception battery

VOSP subtest	5% cut-off score	Test score	Pass/fail
Object perception			

Screening test	15	20	Pass
Incomplete letters	17	19	Pass
Silhouettes	16	23	Pass
Object decision	15	18	Pass
Progressive silhouettes	14	12	Pass

Space perception			

Dot counting	8	9	Pass
Position discrimination	18	20	Pass
Number location	7	4	Fail
Cube analysis	6	10	Pass
